# Intestinal Translocation of Clinical Isolates of Vancomycin-Resistant *Enterococcus faecalis* and ESBL-Producing *Escherichia coli* in a Rat Model of Bacterial Colonization and Liver Ischemia/Reperfusion Injury

**DOI:** 10.1371/journal.pone.0108453

**Published:** 2014-09-25

**Authors:** Karin M. van der Heijden, Inneke M. van der Heijden, Flavio H. Galvao, Camila G. Lopes, Silvia F. Costa, Edson Abdala, Luiz A. D’Albuquerque, Anna S. Levin

**Affiliations:** 1 Department of Infectious Diseases, LIM 54, Hospital das Clínicas, University of São Paulo, São Paulo, Brazil; 2 Transplantation Division, Department of Gastroenterology, University of São Paulo, São Paulo, Brazil; 3 Instituto de Medicina Tropical, Universidade de São Paulo, São Paulo, Brazil; Charité, Campus Benjamin Franklin, Germany

## Abstract

The objectives of this study were to develop a rat model of gastrointestinal colonization with vancomycin-resistant *Enterococcus faecalis* (VRE) and extended-spectrum beta-lactamase (ESBL)-producing *E. coli* and to evaluate intestinal translocation to blood and tissues after total and partial hepatic ischemia. **Methods** - We developed a model of rat colonization with VRE and ESBL-*E coli*. Then we studied four groups of colonized rats: **Group I** (with hepatic pedicle occlusion causing complete liver ischemia and intestinal stasis); **Group II** (with partial liver ischemia without intestinal stasis); **Group III** (surgical manipulation without hepatic ischemia or intestinal stasis); **Group IV** (anesthetized without surgical manipulation). After sacrifice, portal and systemic blood, large intestine, small intestine, spleen, liver, lungs, and cervical and mesenteric lymph nodes were cultured. Endotoxin concentrations in portal and systemic blood were determined. **Results** – The best inocula were: VRE: 2.4×10^10^ cfu and ESBL-*E. coli:* 1.12×10^10^ cfu. The best results occurred 24 hours after inoculation and antibiotic doses of 750 µg/mL of water for vancomycin and 2.1 mg/mL for ceftriaxone. There was a significantly higher proportion of positive cultures for ESBL-*E. coli* in the lungs in Groups I, II and III when compared with Group IV (67%; 60%; 75% and 13%, respectively; p:0.04). VRE growth was more frequent in mesenteric lymph nodes for Groups I (67%) and III (38%) than for Groups II (13%) and IV (none) (p:0.002)**.** LPS was significantly higher in systemic blood of Group I (9.761±13.804 EU/mL−p:0.01). No differences for endotoxin occurred in portal blood. **Conclusion** –We developed a model of rats colonized with resistant bacteria useful to study intestinal translocation. Translocation occurred in surgical procedures with and without hepatic ischemia-reperfusion and probably occurred via the bloodstream. Translocation was probably lymphatic in the ischemia-reperfusion groups. Systemic blood endotoxin levels were higher in the group with complete hepatic ischemia.

## Introduction

Bacterial translocation is a harmful complication of a large number of gastrointestinal disorders including cirrhosis, liver and intestinal ischemic injury and inflammatory bowel diseases.

In several situations, including ischemia and reperfusion lesion, changes in the barrier function of the intestinal epithelium may predispose to increased intestinal permeability, enabling the passage of food, antigens and luminal bacteria [1]–[5].

The ischemia and reperfusion lesion is characterized by a variable period of visceral ischemia with an increase of injury after revascularization [6].

Liver ischemia and reperfusion may result in severe impairment of the intestinal microcirculation. Potential sequelae are: mucosal damage, loss of intestinal barrier function, bacterial translocation, systemic inflammation, multiple organ failure and death [7].

The majority of translocated bacteria are Gram-negative and come from the normal gut flora, suggesting a breakdown of the intestinal barrier function [8], [9].

Intestinal bacteria were correlated as agents in septic patients, suggesting that the gut can be a possible reservoir for bacteria that cause systemic infection by translocation [10], [11].

*Enterococcus* and *Enterobacteriaceae* are involved in bacterial translocation [12] in clinical situations; however the mechanism of bacterial migration is still poorly understood. Several studies support the hypothesis that bacterial translocation is responsible for the dissemination of these bacteria, but few researchers could demonstrate the kinetics, to prove that the bacteria detected in blood and sterile sites has actually originated from the gastrointestinal tract of animals and of humans [1], [10], [11], [13], [14]. There is a lack of models to study the kinetics of *Enterococcus* and *Enterobacteriaceae* migration during bacterial translocation.

The objectives of this study were:

1. To develop an innovative rat model of colonization by resistant *Enterococcus faecalis* and extended-spectrum beta-lactamase (ESBL)-producing *E. coli* of the gastrointestinal tract (GIT).
2. To analyze the profile of intestinal translocation of these bacteria to blood (portal and systemic), lung, liver, cervical and mesenteric lymphnode and spleen after liver ischemia/reperfusion injury with or without intestinal venous stasis.

## Methods

### Developing the model of colonization (Objective 1)

We used Males Wistar rats (*Ratus norvergicus*) from the University of São Paulo School of Medicine animal facility, weighing between 300 and 350 g, housed individually in specific pathogen-free environment in cages with wide wire-mesh bottoms to reduce coprophagy and fed with rodent chow and water *ad libitum*. Animals were acclimated to their environment for 7 days before the experiments. The vivarium room was kept on a 12-hour light/dark cycle, room temperature was maintained at 20±2°C, and humidity ranged around 50%. The colonization process occurred in three phases: decolonization of original intestinal flora, inoculation with the predefined bacteria and determination of the ideal time for maximal colonization after the inoculation.

Ten protocols were tested, each with 4 or 5 rats. We adjusted the antibiotic doses and the bacterial concentrations according to the results of the microbiological growth in the feces. Our intention was to inhibit the growth of the naive intestinal microorganisms allowing only the growth of the inoculated bacteria. In Table 1 we show the doses of antibiotics and inoculums (bacteria concentration) used in each protocol. The animals received drinking water containing vancomycin [15] and ceftriaxone [16] for 7 days prior to the inoculation of bacteria and throughout the duration of the experiment. We modified the concentrations of bacteria in the suspension for each pilot protocol until the ideal above concentrations were achieved.

**Table 1:** Results of stool culture obtained from rats submitted to 10 different protocols of colonization with extended-spectrum beta-lactamase-producing *E. coli* and vancomycin-resistant *E. faecalis*, after an initial decolonization phase using antibiotics.

	number of rats	Antibiotic concentrationsin drinking water		Bacterialinoculum(cfu/animal)		Number of rats withpositive culture					
		Vancomycin (µg/mL)	Ceftriaxone (mg/mL)			24 hours		48 hours		72 hours	
Protocol				VRE	EC	VRE	EC	VRE	EC	VRE	EC
1	4	250	1.4	2×10^8^	2×10^8^	0	0	1	0	1	0
2	4	500	1.4	4.8×10^8^	4.4×10^8^	2	0	0	0	4	0
3	5	750	3.5	10.4×10^8^	12×10^8^	4	0	2	0	4	0
4	5	750	3.5	12×10^8^	24×10^8^	5	0	1	0	1	0
5	4	750	0	12×10^8^	24×10^8^	0	0	0	0	0	0
6	4	0	3.5	1.8×10^10^	3.6×10^9^	0	0	0	0	0	0
7	6	750	0	1.8×10^10^	3.6×10^9^	0	0	0	0	0	0
8	4	750	3.5	1.8×10^10^	3.6×10^9^	4	0	0	0	2	0
9	4	750	2.1	2.28×10^10^	1.08×10^10^	3	4	2	3	1	0
10	4	750	2.1	2.4×10^10^	1.12×10^10^	4	4	3	2	2	1

VRE: Vancomycin-resistant *Enterococcus faecalis*; EC: Extended-spectrum beta-lactamase-producing *Escherichia coli;* cfu: colony-forming units.

We used an isolate of vancomycin-resistant *E. faecalis* (VRE), with a minimal inhibitory concentration (MIC) of 64 µg/mL to vancomycin, and an extended-spectrum beta-lactamase-producing *E. coli* (ESBL-*E. coli*), with a MIC of 128 µg/mL to ceftriaxone. Both isolates were obtained from clinical specimens of patients hospitalized in Hospital das Clínicas, a tertiary-care teaching hospital affiliated to the University of São Paulo. The microorganisms were cultured on sheep agar-blood plates and stocked cultures were prepared incubating the organism overnight in Brain Heart Infusion broth (BHI) at 35°C. The minimum inhibition concentrations (MIC) for each strain were determined following the CLSI criteria [17].

Following the gavage with bacterial suspension, we collected fresh fecal pellets at 24, 48 and 72 hours and processed them according to conventional microbiological methods [18], [19]. The optimum time of colonization was established as the time in which bacterial growth occurred in all the animals.

Additionally, cultures were performed of stools before decolonization with antibiotics and also after the antibiotics before the bacterial gavage.

#### Microbiologic and typing methods

Two grams of feces collected in a dry and sterile bottle were placed in a tube containing BHI broth (Brain Heart Infusion, Oxoid, Hampshire, England) and incubated at 35°C for 18–24 hours in aerobiosis (34). After this period, we performed a subculture of 50 µL of BHI broth placed on sheep blood agar (Oxoid, Hampshire, England), MacConkey agar and SS agar (*Salmonella-Shigella,* Oxoid, Hampshire, England) and analyzed macroscopically and microscopically the bacterial growth. We identified the isolates by classical biochemical tests [18]. We subjected the isolates of *E. coli* to screening in selective media Mueller-Hinton agar with 100 mg of ceftriaxone [17]. The resistant isolates, with a minimal inhibitory concentration (MIC) superior to 100 mg/mL were submitted to double disc test for confirmation of ESBL production. We performed the VRE screening test using a BHI plate containing 6 mg/mL of vancomycin [17]. *E. coli* ATCC 25922 and *E. faecalis* ATCC 29212 were used as control strains.

*E. coli* and *E. faecalis* stool isolates were submitted to molecular analysis (pulsed field gel electrophoresis – PFGE) as described elsewhere [20], [21] and the molecular patterns with the strains used in the gavage were compared.

### Evaluation of bacterial translocation (Objective 2)

The rats received drinking water containing vancomycin (750 µg/mL) and ceftriaxone (2.1 mg/mL) and throughout the duration of the experiment. On the eighth antibiotic day, we inoculated ESBL-*E. coli* and VRE via gavage, based on the results of the experiments described above. Thus, on the eight day of antibiotic use (day zero) each animal received 6 mL of bacterial suspension via gavage composed of half containing 2.4×10^10^ colony-forming units (CFU) of VRE and half containing 1.12×10^10^CFU of ESBL*-E. coli.*

The rats were divided into four groups: **Group I** (n = 15) rats submitted for 30 minutes to hepatic pedicle occlusion (portal vein and hepatic artery) causing complete liver ischemia and intestinal stasis and sacrificed after 1 hour of reperfusion. **Group II** (n = 15) rats submitted for 30 minutes to partial liver ischemia (pedicle occlusion of median and left lateral hepatic lobes) without intestinal stasis and sacrificed after 1 hour of reperfusion. **Group III** (n = 8) rats that suffered the same surgical manipulation as previously described without hepatic ischemia or intestinal stasis (SHAM group) during 90 minutes. **Group IV** (n = 8) rats anesthetized during 90 minutes without surgical manipulation (non-surgical control group). All surgeries were performed under anesthesia, and all efforts were made to minimize suffering.

The rats were anesthetized intramuscularly with an association of 75 mg/kg of ketamine hydrochloride 5% (Ketalar, Cristália, São Paulo, Brazil) and 10 mg/kg of xylazine (Dorcipec, Vallé, Minas Gerais, Brasil). We kept the animals at a body temperature of 35–37°C throughout the procedure and performed a median laparotomy. Rats from Group I were submitted to the hepatic pedicle occlusion (portal vein and hepatic artery) with microvascular atraumatic clamps causing complete liver ischemia and intestinal stasis (congestive ischemia).

At the time of the sacrifice, we cultured samples from portal and systemic blood, large intestine, small intestine, spleen, liver, lungs, and cervical and mesenteric lymph nodes. We identified VRE and ESBL-*E. coli* by the profile of resistance. Isolates were submitted to molecular typing by pulsed-field gel electrophoresis (PFGE). We determined endotoxin concentrations in plasma from both portal and systemic blood by a kinetic and quantitative assay using Limulus Amebocyte Lysate kit.

All experimental procedures were in accordance with the National Council of Control of Animal Experimentation guidelines for the care and use of laboratory animals. The animal protocol used in the present study was reviewed and approved based on ethical procedures and scientific care by the Committee on the Ethics in Research of the University of São Paulo School of Medicine (CAPPesq protocol number 004/04).

#### Collection of specimens

The collection of material in groups I and II, was performed 1 hour after liver and intestinal reperfusion, and in the control groups III and IV after a similar period. For all groups, we collected 3 mL of portal and systemic blood for endotoxin measurement and cultures. The blood was immediately inoculated in *culture bottles* (Bac-hemocult pediátrico®, DME, Araçatuba, Brazil), incubated at 35°C for 30 days and grown on sheep blood agar 5%.

Rats’ necropsies: macerated fragments of large intestine, small intestine, spleen, liver, lungs, and cervical and mesenteric lymph nodes were inoculated into brain-heart agar (BHI) broth at 35°C. After 24 hours, 0.01 mL of this broth was inoculated on blood agar plates and 0.1 mL in selective broth for VRE and *E. coli* resistant to ceftriaxone (MIC>100 mg/mL). The stocks were plated on blood agar and incubated at 35°C±2°C.

#### Molecular bacterial typing

In order to confirm that the bacteria isolated originated from the inoculated strain, we submitted the isolates to molecular analysis by pulsed-field gel electrophoresis (PFGE) as described elsewhere [20], [21].

#### Endotoxin assay

We determined endotoxin concentrations in plasma obtained from portal and systemic blood by a kinetic and quantitative assay, Limulus Amebocyte Lysate kit (LAL - Pyrogent-5000, Cambrex Bio Science, Walkersville, USA). Diluted plasma samples (1∶100) were mixed with reconstituted LAL reagent, placed in photometer and automatically monitored until appearance of turbidity. The time required before turbidity (reaction time) is inversely proportional to the amount of endotoxin. We obtained a standard endotoxin curve (0.01 to 100 EU/mL) with *Escherichia coli* reference endotoxin (*E. coli* O55:B5) according to the manufacturer's instructions and calculated it based in the concentrations of endotoxin within unknown samples on this standard curve. The samples were suspended in 1 mL of LAL water supplied by the kit and agitated in vortex for 60 seconds. Immediately afterwards, we added 100 µL of the blank, followed by the same volume of the standard endotoxin solutions (0.01, 0.1, 1, 10, 100 EU/mL) and 100 µL of the plasma diluted samples in duplicate to the 96-well microplate with their respective controls. We incubated these mixes for 10 min at 37°C±1°C and added 0.1 mL of PYROGENT-5000 reagent (previously reconstituted). We gently shook the microplates and the absorbance was read. The microplate reader/WinKQCL Software (BioWittaker, Cambrex Co, Walkersville, MD) monitored the absorbance at 340 nm of each well of the microplate continuously throughout the assay. The WinKQCL Software automatically performed a log/log linear correlation of the reaction time of each standard with its corresponding endotoxin concentration and printed the standard curve parameters. We performed all the controls according to manufacturer's instructions. The absolute value of the correlation coefficient of standard curve was greater than or equal to 0980. The reaction occurred within the limits of 0.00 to 100 EU/ml.

#### Statistical analysis

We determined the proportion of animals showing positive for the strains of ESBL *E. coli* and VRE in the blood or any other tissue. The mean, median and standard deviation of endotoxin levels in portal and systemic blood were quantified. We compared the proportion of animals with positive cultures for organs or tissue in each group using the chi-square test of Person. The groups that presented statistically significant positive cultures for *E. coli* and VRE were submitted, two by two, to a comparative analysis. The analyses between groups were made by Fisheŕs test.

Concentrations of endotoxin for each group were compared using the ANOVA test. The cases that showed statistically significant differences were submitted, two by two, to a comparative analysis using Tukey test.

The level of significance was 5% for all tests performed using the software IBM SPSS 13 for Windows (SPSS, Chicago, IL, USA).

## Results

### Developing the model of colonization

In Table 2 we describe the predominant bacteria found before and after the use of antibiotics (before bacterial gavage).

**Table 2:** Microbiological results of stool culture obtained from rats before and after decolonization with antibiotics (vancomycin and ceftriaxone).

	Number and proportionof positive cultures	
	Before using antibiotics n: 42	After decolonization using antibiotics n: 42
*E. coli*	38 (90%)	3 (7%)
*Enterococcus* spp.	27 (64%)	17 (40%)
*Staphylococcus* spp.	11 (26%)	10 (24%)
*Streptococcus* spp.	23 (55%)	2 (5%)
*Proteus* spp.	19 (45%)	0
*Edwardsiella* spp.	5 (12%)	0

Table 1 summarizes the antibiotic doses, VRE and ESBL-*E. coli* inoculums, and the proportion of rats with growth from stool culture at 24, 48 and 72 hours. The best inoculum of VRE was 2.4×10^10^ cfu and of ESBL-*E. coli* was 1.12×10^10^ cfu. The best results were encountered 24 hours after inoculation and the best antibiotic doses were 750 µg/mL of water for vancomycin and 2.1 mg/mL of water for ceftriaxone. Based on the usual rat daily drinking intake of 15 mL we estimated the daily dose of vancomycin to be 32 mg/Kg and of ceftriaxone to be 90 mg/Kg. According to the PFGE, VRE and ESBL-*E. coli* obtained from the stools of the rats were identical to the strains inoculated by gavage.

### Evaluation of bacterial translocation

Tables 3 and 4 summarize the results of microbiological growth for strains of ESBL *E. coli* and VRE in different organs and tissues. Considering the growth of ESBL-*E coli* there was a significantly higher proportion of positive cultures in the lungs of rats in Groups I, II and III when compared with the non-surgical control group (Group IV). There was more VRE growth in mesenteric lymph nodes for groups I and III. In Table 5 we show the serum endotoxin levels in the portal and systemic blood for each group. There was no statistically significant difference between the groups when comparing the concentration of endotoxin levels in portal blood. However the levels of endotoxin in systemic blood were significantly higher in the hepatic ischemia groups (Groups I and II) than in the controls (groups III and IV). Molecular typing demonstrated that the ESBL-*E. coli* and VRE isolates from organs and tissues were identical to the isolates used to colonize the GIT of the rats.

**Table 3:** Number and proportion of rats with positive microbiological growth for strains of extended-spectrum beta-lactamase producing *E. coli* in organs and tissues.

Organ/Tissue	Groups				
	I	II	III	IV	P
	n = 15	n = 15	n = 8	n = 8	
Lung	10 (67%)	9 (60%)	6 (75%)	1 (13%)	**0.04** *
Spleen	3 (20%)	3 (20%)	2 (25%)	0	0.54
Liver	8 (53%)	8 (53%)	6 (75%)	3 (38%)	0.51
Portal Blood	2/12 (17%)	1 (6%)	0/7	1 (13%)	0.64
Systemic Blood	3/12 (25%)	1 (6%)	1/7 (14%)	0	0.32
Mesenteric lymph nodes	7 (47%)	3 (20%)	4 (50%)	1 (13%)	0.17
Cervical lymph nodes	3 (20%)	4 (27%)	1 (13%)	1 (13%)	0.80

I - surgical group with hepatic ischemia and intestinal stasis; II - surgical group with hepatic ischemia without intestinal stasis; III - surgical control group; IV - non-surgical control group.

*Fisher’s test comparing groups: I and IV p:0.02; II and IV p:0.04; III and IV p:0.02.

**Table 4:** Number and proportion of rats with positive microbiological growth for strains of vancomycin-resistant *Enterococcus* in organs or tissues.

	Groups				
Organ/Tissue	I	II	III	IV	P
	n = 15	n = 15	n = 8	n = 8	
**Lung**	11 (73%)	11 (73%)	5 (63%)	3 (38%)	0.31
**Spleen**	6 (40%)	5 (33%)	2 (25%)	0	0.22
**Liver**	11 (73%)	8 (53%)	8 (100%)	3 (38%)	**0.04** *
**Portal Blood**	2/12 (17%)	1 (6%)	0/7	1 (13%)	0.65
**Systemic Blood**	2/12 (17%)	0	0	0	0.14
**Mesenteric lymph nodes**	10 (67%)	2 (13%)	3 (38%)	0	**0.002** **
**Cervical lymph nodes**	5/12 (33%)	4 (27%)	1 (13%)	1 (13%)	0.59

I - surgical group with hepatic ischemia and intestinal stasis; II - surgical group with hepatic ischemia without intestinal stasis; III - surgical control group; IV - non-surgical control group.

*Fisher’s test comparing groups: II and III p:0.03; III and IV p:0.01.

**Fisher’s test comparing groups: I and IV p:0.003; I and II p:0.004.

**Table 5:** Concentration of endotoxin in the portal and systemic blood of rats submitted to surgical procedures or control.

	Groups					
		I	II	III	IV	P*
		n = 15	n = 15	n = 8	n = 8	
Mean**(**Standard deviation)EU/mL	PortalBlood	1.868 (3.230)	0.776 (1.322)	0.121 (0.321)	0.280 (0.737)	0.24
	SystemicBlood	9.761 (13.804)	1.425 (2.179)	0.146 (0.385)	0.018 (0.051)	**0.01** *
Median	PortalBlood	0.650	0	0	0	
	SystemicBlood	1.605	0.420	0	0	

I - surgical group with hepatic ischemia and intestinal stasis; II - surgical group with hepatic ischemia without intestinal stasis; III - surgical control group; IV - non-surgical control group.

*Fisher’s test comparing groups: I and II p:0.03; I and III p:0.03; I and IV p:0.03.

## Discussion

In this study we describe an original and efficient experimental model of GIT colonization in rats using as a marker to evaluate translocation antimicrobial resistance in bacteria (ESBL-producing *E. coli* and vancomycin-resistant *E. faecalis*).

When evaluating protocols 5 and 7 (Table 1), in which the rats did not receive ceftriaxone in contrast with protocols 4 and 8, respectively, the growth of VRE did not occur. The use of ceftriaxone seemed to have had an effect on VRE colonization. There are epidemiologic studies showing an association between the previous use of cephalosporins [22], [23], especially ceftriaxone [24], and VRE infection. The explanation for this finding is not obvious but, as discussed by McKinnell et al [24], ceftriaxone is thought to significantly change intestinal flora, reducing microecologic competition and allowing for VRE overgrowth and heavy colonization.

The phenomenon of ischemia and reperfusion (IR) occurs during liver surgeries, including transplantation [25], [26]. IR leads to hepatic injury with functional and structural damage of hepatic cells [27]. The extent of the injury is proportional to the duration of ischemia [28]. Secondary lesions are observed within 60 minutes of reperfusion. In our rat model we studied 2 groups in which ischemia was complete (group I) or partial (group II) and we used 2 control groups: one group in which surgery was performed without ischemia (group III) and one control group without surgery (group IV). Sterile organs and tissues presented the resistant bacteria in a similar proportion for all the surgical groups, suggesting that surgical trauma is enough to cause translocation.

Portal and systemic blood had a lower yield than the cultures of organs and tissues. There are two possibilities to explain this result. Translocation can occur via the lymphatic system and/or the blood stream [13]. One possibility is that the translocation was predominantly lymphatic, thus blood cultures were not sensitive to detect it. Another possibility is that hematogenous translocation was an early event and the blood cultures collected 60 minutes after reperfusion “missed it”. This second explanation is in disaccord with the work of Ren *et al* [29] that observed a higher positivity of blood cultures collected 24 hours after a model of intestinal ischemia/reperfusion injury, but there may have been methodological differences between the two experiments. Translocation through the lymphatic system would allow the bacteria to be initially filtered by the mesenteric lymph nodes resulting in a high proportion of positive cultures such as occurred with VRE in our complete ischemia group (group I). Hematogenous translocation would lead to initial filtration of bacteria by the liver which would cause a high positivity of cultures, as occurred in all our surgical groups. It is possible that the surgical stress led to hematogenous translocation and hepatic ischemia-reperfusion increased lymphatic translocation.

The use of broad spectrum antimicrobials has been shown in experimental studies to be a predisposing factor in itself for translocation of vancomycin-resistant enterococci [30], [31]. This may, in part, explain the translocation that was observed in group IV, in which the rats had not been submitted to surgical procedures.

Endotoxin levels in the systemic blood were significantly higher in the rats submitted to complete ischemia (Group I). This is probably explained by a higher release of intestinal endotoxin in the animals with intestinal stasis, associated with a lower hepatic clearance caused by IR. Probably the rats in Group II presented higher bacterial clearance capacity of the liver due to only partial ischemia.

Markers to study translocation include bacteria marked with radioactive isotopes [32], [33] which make studies difficult to perform. The use of genomic techniques to study the microbiome may not be satisfactory in evaluating translocation because typing is necessary to evaluate the source [29]. Our colonization model proved to be a low-cost, useful and reproducible tool to study translocation because resistance was used as a marker that can be identified by relatively simple phenotypic methods.

Our study has limitations. We did not use quantitative cultures when developing the model of colonization and when evaluating translocation to the tissues. We preferred to use the proportion of colonized rats to define proportion of colonization and the proportion of positive tissue cultures to determine frequency of translocation. We also did not use sequencing methods to evaluate the microbiota of the GIT of the rats previous to and during the experiments, although these molecular methods potentially could have enhanced the bacterial diversity observed. *Enterococcus* spp. was present in 40% of rats before colonization with VRE. This may have affected colonization and led to less translocation of VRE. If so, our rates of translocation may have been underestimated.

In conclusion, we developed a model of rats colonized with resistant bacteria which may be useful to study intestinal translocation. Translocation occurred in all surgical procedures with and without hepatic ischemia-reperfusion and probably occurs predominantly via the bloodstream. Translocation probably occurred through the lymphatic system in the ischemia-reperfusion groups. Systemic blood endotoxin levels were higher in the group with complete hepatic ischemia.
